# Editorial: Community series in genetic mechanisms of biomarkers in schizophrenia, bipolar disorder and depression, volume II

**DOI:** 10.3389/fpsyt.2023.1267708

**Published:** 2023-11-16

**Authors:** Yubing Yin, Shaohua Hu, Sarah Tarbox-Berry, Qiang Wang

**Affiliations:** ^1^Mental Health Center and Psychiatric Laboratory, State Key Laboratory of Biotherapy, West China Hospital of Sichuan University, Chengdu, Sichuan, China; ^2^West China Brain Research Center, West China Hospital of Sichuan University, Chengdu, Sichuan, China; ^3^Sichuan Clinical Medical Research Center for Mental Disorders, Chengdu, Sichuan, China; ^4^Department of Psychiatry, First Affiliated Hospital, Zhejiang University School of Medicine, Hangzhou, Zhejiang, China; ^5^Department of Neurology, School of Medicine, Wayne State University, Detroit, MI, United States; ^6^Department of Psychiatry, School of Medicine, Yale University, New Haven, CT, United States

**Keywords:** genetic mechanism, biomarker, schizophrenia, bipolar disorder, depression

Schizophrenia, bipolar disorder, and depression are three major psychiatric disorders that significantly contribute to morbidity, mortality, and societal burden (1). Despite the profound impact they pose, the exact etiology of these disorders remains elusive, and the diagnoses are made based on symptom-based criteria in International Classification of Diseases (11th revision, ICD-11) and the Diagnostic and Statistical Manual of Mental Disorders (5th edition, DSM-5). Consequently, research into the genetic mechanisms and biomarkers of these disorders is of critical importance.

The second edition of this Research Topic aims to further our understanding of the genetic mechanisms and biomarkers underlying schizophrenia, bipolar disorder, and depression. This edition comprises eight articles, covering a diverse range of objectives and methodologies, including genetic research, investigations into functional brain alterations in these disorders, and a bibliometric study.

Schizophrenia, bipolar disorder, and depression are disorders with relatively high heritability. Although previous studies have indeed discovered susceptibility loci from numerous candidate genes (2–4), these findings have not consistently produced verifiable outcomes. Several studies within this Research Topic aim to detect genetic variations related to these disorders. For instance, Tsai et al. performed a Genome-Wide Association Study (GWAS) on the Han Chinese population residing in Taiwan, leading to the discovery that single nucleotide polymorphism variants of the CDH4 intron region (rs78063755), the NTRK3-AS1 downstream region (rs57729223), and between LINC01918 and GPR45 (rs2679891) are suggestively associated with depression. A systematic review by Wang Y. et al. confirmed through a meta-analysis that the BDNF Val66Met polymorphism is a susceptibility factor for Major Depressive Disorder (MDD) in Caucasian populations. Several studies also employed innovative statistical methods. For example, Wang Z. et al. utilized weighted gene co-expression network analysis and machine learning algorithms to identified S100A12, SERPINB2, TIGIT, GRB10, and LHFPL2 in peripheral serum as biomarkers for depression.

Some studies have unveiled findings beyond genetic susceptibility loci. For instance, Xu et al. implemented a machine learning predictive model and found 11 circadian rhythm gene polymorphisms associated with the conversion from major depressive disorder to bipolar disorder (MDD-to-BD) via feature screening. Moreover, they found that factors such as the age of onset, suicide attempts, and the number of hospitalizations also posed risks for the conversion from MDD to BD. This finding holds potential utility in focusing early attention on specific depressive patients to predict their transition to bipolar disorder.

In addition to studies examining individual disorders, cross-diagnostic research is also extremely important. From a clinical perspective, patients with these disorders can sometimes present similar symptoms. In terms of genetic mechanisms, studies have identified multiple overlapping risk genes across these disorders (5). Here, Yu et al. carried out a two-sample Mendelian randomization study examining the effects of plasma homocysteine (Hcy) levels on the risk of these three major psychiatric disorders. They found that a genetic predisposition to elevated plasma Hcy levels was causally linked to an increased risk of schizophrenia (SCZ) and Bipolar I disorder (BD-I), while there was no evidence supporting causal relationship between plasma Hcy levels and major depressive disorder (MDD) and Bipolar II disorder (BD-II).

Apart from genetic research, neuroimaging approaches also hold a prominent place in the field of mental disorders. For example, Sun et al. investigated the resting-state whole-brain dynamical pattern in bipolar disorder patients with different mood states. Their findings illustrated distinct and shared brain dynamical patterns among depressive, manic, and euthymic states. Similarly, Song et al. explored resting functional connectivity (FC) of each hippocampal subregion among patients with schizophrenia, bipolar disorder, and major depressive disorder. They found both shared and uniquely altered functional connectivity within hippocampal subregions across these three disorders.

Lastly, Lam et al. conducted a bibliometric analysis on 8,221 publications, demonstrating rapid growth of research pertaining to the relationship between heart failure and depression. Their work also highlighted key areas for future research, such as self-care practices and anxiety related to heart failure.

In conclusion, diving into the genetic underpinnings of biomarkers associated with schizophrenia, bipolar disorder, and depression enlightens us about the root causes and overlaps in these mental illnesses. As we expand our knowledge in this specific scientific domain, mental health professionals will be better positioned to determine diagnoses, select suitable treatments, and anticipate the trajectory of these conditions.

## Author contributions

YY: Writing – original draft. SH: Writing – review & editing. ST-B: Writing – review & editing. QW: Writing – review & editing.
